# Amorphous Solid Dispersions: Role of the Polymer and Its Importance in Physical Stability and In Vitro Performance

**DOI:** 10.3390/pharmaceutics14081747

**Published:** 2022-08-22

**Authors:** Qin Shi, Haibiao Chen, Yanan Wang, Ruoxun Wang, Jia Xu, Chen Zhang

**Affiliations:** 1 School of Pharmacy, Jiangsu Vocational College of Medicine, Yancheng 224005, China; 2 Institute of Marine Biomedicine, Shenzhen Polytechnic, Shenzhen 518055, China

**Keywords:** amorphous solid dispersion, polymer, physical stability, in vitro performance

## Abstract

Amorphous solid dispersions stabilized by one or more polymer(s) have been widely used for delivering amorphous drugs with poor water solubilities, and they have gained great market success. Polymer selection is important for preparing robust amorphous solid dispersions, and considerations should be given as to how the critical attributes of a polymer can enhance the physical stability, and the in vitro and in vivo performances of a drug. This article provides a comprehensive overview for recent developments in the understanding the role of polymers in amorphous solid dispersions from the aspects of nucleation, crystal growth, overall crystallization, miscibility, phase separation, dissolution, and supersaturation. The critical properties of polymers affecting the physical stability and the in vitro performance of amorphous solid dispersions are also highlighted. Moreover, a perspective regarding the current research gaps and novel research directions for better understanding the role of the polymer is provided. This review will provide guidance for the rational design of polymer-based amorphous pharmaceutical solids with desired physicochemical properties from the perspective of physical stability and in vitro performance.

## 1. Introduction

Amorphization can effectively enhance the solubility and dissolution of active pharmaceutical ingredients (APIs) in water, and it has been successfully applied in delivering poorly water-soluble drugs [1,2,3,4]. However, the physical stability of the drug is compromised due to the higher free energy and the long-range disordered molecular packing of the amorphous phase. As a result, crystallization and phase separation sometimes occur during the manufacturing or storage of amorphous drug formulations [5,6]. To combat the stability issue, the most widely adopted strategy is to molecularly disperse the amorphous drug into a polymer matrix to form an amorphous solid dispersion (ASD) [5,6,7]. Polymer-based ASD formulations have proven their superiority in enhancing the bioavailability of poorly water-soluble drugs, and thus they have attracted considerable interests from pharmaceutical companies and have gained commercial and clinical success over the past decades [8].

The effects of the polymer matrix on the physical stability, in vitro dissolution, retention of supersaturation, and in vivo performance of ASDs have been intensively studied over the past decades [4,5,9,10,11,12,13]. While tremendous efforts have been expended in the developments of ASDs, it should be noted that in practice, only a small number of polymers can be used for preparing ASD formulations. Several mechanisms have been proposed to explain how the behaviors of the polymer during nucleation, crystal growth, and phase separation affect the physical stability of the ASD [5]. Critical properties of the polymer matrix, including the glass transition temperature (*T*_g_), molecular weight, segmental mobility, and the ability to form drug–polymer interactions have been explored, and their correlations with the physical stability of ASD have been established [9,14,15,16,17]. In this review, the first part is to provide a comprehensive overview of the recent advances on exploring the effects of the polymer on maintaining the physical stability of amorphous drugs. 

The effects of the polymer on the in vitro dissolution and supersaturation of ASD have also been extensively studied [3,4]. It is well accepted that the general mechanism behind the enhanced dissolution of an ASD is the so-called “spring and parachute” effect [4]. Achieving and maintaining the supersaturation of an ASD can translate to enhanced permeability through the membrane, which greatly improves oral bioavailability [18]. The second part of this article gives a comprehensive overview of the studies focusing on the dissolution and supersaturation of ASDs. Especially, the effects of the polymer matrix on governing the dissolution and maintaining supersaturation are systemically summarized. This review will also discuss the current research gaps and potential research topics for a better understanding of the role of the polymer in ASD.

## 2. Role of the Polymer in Physical Stability

### 2.1. Effects of the Polymer on Nucleation

Nucleation, the initial stage of crystallization, is a crucial factor in governing the physical stability of amorphous pharmaceutical formulations. During the nucleation stage, nuclei are first born and then grow to a macroscopic size. Nucleation has been explained via several mechanisms, including classical nucleation theory (CNT), secondary nucleation theory, etc. [5,19,20,21,22]. According to CNT, the steady state rate of the homogeneous nucleation of a one-component system can be described using the following expression.

(1) $$J=K_{J}\exp\left(\frac{-W_{c}}{kT}\right)$$

(2) $$W_{c}=\frac{16\pi }{3}\frac{\sigma^{3}}{\Delta G_{V}^{2}}$$

Herein, *W_c_* is the thermodynamic barrier for the creation of a critical nucleus. Δ*G_v_* is free energy difference between the crystal and the liquid, which can be calculated from the Hoffman equation as follows;

(3) $$\Delta G_{v}=\frac{\Delta H_{f}\left(T_{m}-T\right)T}{T_{m}^{2}}$$

Herein, Δ*H_f_* represents the heat of fusion and *T_m_* is the melting point, which can be obtained via differential scanning calorimetry (DSC). *σ* represents nucleus–liquid interfacial free energy, which is generally not precisely measured from independent measurements or simulations. Given that a small variation in σ would lead to orders of magnitude in the nucleation rate *J*, Equation (1) generally acts as a fitting model, rather than a predictive one. *K_J_* is a kinetic factor representing the attempt frequency of molecules entering into the nucleus. The kinetic factor *K_J_* is commonly expressed as a function of the measurable liquid dynamic parameters, including α-relaxation time, viscosity, and bulk diffusion coefficient. 

According to CNT, the major factors affecting the nucleation process include the thermodynamic driving force, molecular mobility, liquid–crystal interfacial energy, and molecular recognition. Via determining the maximal rate of nucleation, the effects of thermodynamic and kinetic factors on the rate of nucleation were found to show opposing temperature dependencies. Recent studies proposed a two-step nucleation mechanism challenging the CNT [21,23]. Initial breeding, dendritic or needle propagation, and attrition have been proposed to the possible origins in the secondary nucleation theory during exploration over the past few decades [21]. However, it should be noted that a current understanding of the nucleation process is still far from perfect, from either the theoretical or experimental perspectives. 

It has been widely reported that polymers can affect the nucleation of amorphous drugs. However, the underlying mechanism of the effects of polymers on drug nucleation is quite controversial in the literature [9,17,24,25,26,27]. Given that the number of nuclei can strongly affect recrystallization, the position of the recrystallization exothermal peak in the differential thermal analysis curve during the heating process of amorphous drugs is proposed to be a reliable indicator for studying the nucleation [17,25]. A recrystallization peak at a higher temperature indicates the formation of fewer nuclei, and vice versa [17,25]. On the basis of this assumption, Trasi et al. investigated the effects of the polymer on the nucleation of amorphous acetaminophen by evaluating the nucleation zone [25]. No further decrease in the nucleation rate was observed with the decrease in temperature once the system entered the nucleation region [25]. Herein, the lower limit of the nucleation zone is defined as being the temperature where further cooling leads to no considerable change in the location and shape of the crystallization peak [25]. Correspondingly, the upper limit of the nucleation zone is defined as the highest temperature where the system is cooled and where recrystallization occurs upon heating [25]. Above the upper limit, the rate of nucleation was extremely low and no detectable level of nucleation was observed during the cooling and heating processes [25]. Above the upper limit, the rate of nucleation was extremely low and no detectable level of nucleation was observed during the cooling and heating processes [25].

**Figure 1:**
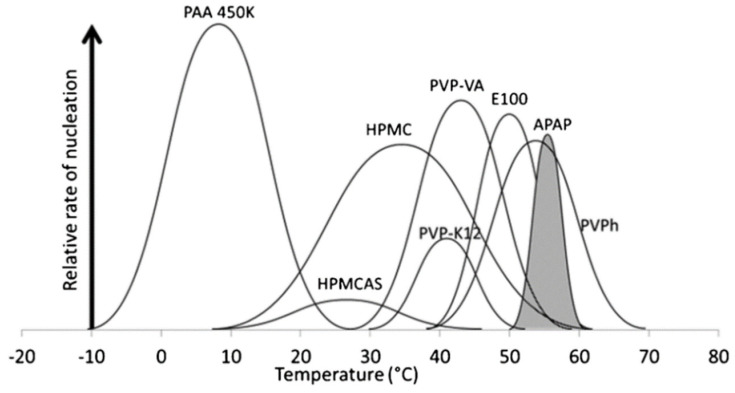
A semi-quantitative representation of the nucleation temperature zones and relative nucleation rates of pure acetaminophen (shaded region) and acetaminophen in the presence of 10% *w*/*w* polymers. Adapted from Ref. [25] with permission. Copyright © 2012 The Royal Society of Chemistry.

Figure 1 shows a semi-quantitative representation of the relative rates and zones of nucleation of acetaminophen in pure and 10% *w*/*w* polymer-doped systems. The average number of nuclei of acetaminophen is not significantly affected by the addition of 10% *w*/*w* hydroxypropyl methylcellulose (HPMC), Eudragit E100, poly(vinyl phenol), or PVP-vinyl acetate (PVPVA) [25]. For comparison, the addition of HPMC-Acetyl succinate (HPMCAS) or poly(vinyl pyrrolidone) (PVP) K12 can effectively inhibit the nucleation of acetaminophen [25]. For instance, HPMCAS exhibits the best inhibitory effect on nucleation and reduces the number of the nuclei of acetaminophen to around a third of the pure drug [25]. Interestingly, poly(acrylic acid) (PAA) is found to accelerate rather than inhibit the nucleation of acetaminophen [25]. Compared to pure acetaminophen, a nearly three-fold number of nuclei are formed with the addition of 10% *w*/*w* PAA [25]. Here, the enhanced nucleation of acetaminophen in a PAA-doped system is proposed to be a result of molecular recognition events. PAA may exhibit a local ordering effect on the nucleation of acetaminophen, which is analogous to its role in solution as a template to induce the formation of particular polymorphs [28]. 

In addition, determining the temperature dependence of nucleation is of great importance. If ASDs are stored at a temperature within the nucleation zone, they can experience nucleation at high rates. For instance, compared to a PVP-doped system, nucleation in HPMCAS-doped ASD is better inhibited [25]. However, if the ASD is to be stored at room temperature, HPMCAS-doped ASD would potentially show a greater number of nuclei in comparison with the PVP-doped system [25]. This result is mainly attributed to the higher nucleation rate in HPMCAS-doped systems in comparison with that in a PVP-doped system at room temperature [25]. 

Recent works also develop one-stage and two-stage methods for determining the nucleation rates of amorphous pharmaceutical solids as a function of temperature [9,26,29,30]. The one-stage and two-stage methods can be used to determine the number of nucleation events per unit volume, respectively, for amorphous systems showing distinct stages with relatively fast and slow crystal growth rates [29]. By applying one-stage and two-stage methods, Huang et al. measure the nucleation rates of four polyalcohols, i.e., D-sorbitol, D-arabitol, D-xylitol, and glycerol [30]. One key finding of this study is that the nucleation rates of these polyalcohols are vastly different as a function of *T*/*T*_g_ [30]. For comparison, the crystal growth rates of these polyalcohols are similar, with a fastest rate near 1.4 *T*_g_ at same *T*/*T*_g_ scale [30]. Here, they proposed that CNT can provide a reasonable description of the nucleation kinetics by considering the crystal growth rate as a kinetic barrier [30]. This postulation is also supported in a very recent study focusing polymorphic selectivity during crystal nucleation [31].

**Figure 2:**
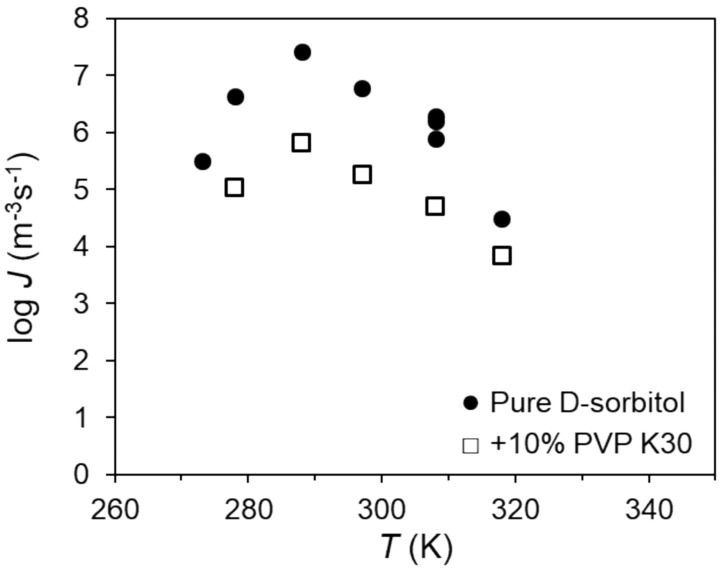
Effect of 10 wt% PVP K30 on the rate of nucleation of D-sorbitol. The solid circle and hollow square respectively represent the nucleation rates of pure D-sorbitol and PVP-doped system measured using two-stage method. Adapted from Ref. [26] with permission. Copyright © 2019 American Chemical Society.

Yao et al. investigate the effects of PVP on the nucleation and crystal growth of two molecular liquids, i.e., D-sorbitol and D-arabitol [26]. The addition of PVP exhibited a significant inhibitory effect on the nucleation of these two molecular liquids [26]. For instance, 10 wt% PVP K30 can slow down the nucleation of D-sorbitol by nearly 40-fold near the peak temperature of nucleation rate (Figure 2) [26]. The inhibitory effect of PVP could be enhanced with an increase in the polymer concentration and molecular weight [26]. Interestingly, the inhibitory effect of the polymer on nucleation has been demonstrated to be nearly the same as that on crystal growth [26]. The ratio between the nucleation rate and the crystal growth rate is nearly a constant that is independent of polymer molecular weight and concentration at a given temperature [26]. These results indicate that both nucleation and crystal growth in a melt are mobility-limited processes, and that the polymer in an amorphous solid mainly acts as a mobility modifier [26]. Similar effects were also observed in the nucleation and crystal growth of nifedipine in the presence of four different surfactants, including Tween 80, Span 80, Triton X-100, and Poloxamer 407. The addition of 10% *w*/*w* surfactants can effectively accelerate drug nucleation and crystal growth by up to two orders of magnitude [32]. These surfactants exhibit similar enhancement effects on crystallization, independent of their molecular structure and hydrophilic-lipophilic balance [32]. These results indicate that surfactant adsorption at the solid–liquid interface is not the major factor affecting the crystallization [32]. 

In a very recent study, Zhang et al. explored the effects of three chemically distinct polymers on the nucleation of a classical antifungal drug fluconazole [9]. A concentration of 10 wt% HPMCAS could strongly inhibit the nucleation of fluconazole, while the same content of PVP exhibited a minor inhibitory effect [9]. For comparison, the addition of 10 wt% poly(ethylene oxide) (PEO) could substantially increase the nucleation rate of fluconazole polymorphs [9]. This enhancement in the nucleation rate of fluconazole polymorphs is proposed to be mainly attributed to the increase in the molecular collision frequency in amorphous fluconazole via the addition of PEO [9]. Moreover, they also find that the kinetics of the nucleation and crystal growth of fluconazole can be influenced by these polymer additives to a similar extent [9]. These results further support the view that nucleation and crystal growth processes share a similar kinetic barrier [9].

### 2.2. Effect of the Polymer on Crystal Growth

Compared to the nucleation process, more attentions have been focused on the crystal growth of amorphous pharmaceutical solids [5,15]. In a supercooled liquid state, the crystal growth of a one-component amorphous system depends on both the molecular mobility and the thermodynamic driving force [5,15]. A bell-shaped curve of crystal growth can be generally observed in the supercooled liquid of a one-component amorphous system [5,15]. Near *T*_m_, the thermodynamic driving force is the main factor controlling the crystal growth of a one-component amorphous system. With an increase in supercooling, the molecular mobility takes the place of the thermodynamic driving force and gradually becomes the rate-limiting factor of crystal growth. It is widely accepted that bulk diffusion controls the crystal growth in the supercooled liquid at temperatures far away from *T*_m_ [33,34]. This notion is strongly supported by the proportionality between the rate of crystal growth and the coefficient of bulk diffusion [34]. According to the bulk diffusion-controlled model, crystal growth is expected to be extremely slow near or below *T*_g_. However, in the past few decades, considerable studies have shown that some organic molecules can lead to a much faster crystal growth in comparison with those predicted using the bulk diffusion-controlled model [35,36,37,38,39]. One phenomenon occurring in the interior of amorphous solids, termed as glass-to-crystal (GC) growth, has been explained using several mechanisms [35,36]. The other one occurring at the free surface is proposed to originate from fast surface diffusion [37,38]. 

Current understanding suggests that polymer additives can strongly affect the crystal growth of amorphous pharmaceutical solids [5,14,16]. One of the key factors affecting crystal growth is the formation of the intermolecular interactions (hydrogen bonding, ionic interaction or dipole–dipole interactions) between the drug and the polymer [25,40,41]. For instance, the inhibitory effect of PVP on the crystallization of amorphous indomethacin (IMC) is attributed to the hydrogen bonding between the carboxylic acid groups of the drug and the carbonyl group of the polymer [41]. Taylor and coworkers investigated the inhibitory effects of several polymers on the crystal growth of an antihypertensive drug felodipine [40]. They reveal that stronger or more extensive drug–polymer hydrogen bonding could translate to a better inhibitory effect on the crystal growth of the drug [40]. Similar correlations between the strength/extent of the drug–polymer hydrogen bonding interaction and the crystal growth kinetics have also been reported in the solid dispersion of acetaminophen [25]. In the case of acetaminophen, whose molecular structure contains both hydrogen bonding donors and acceptors, a polymer containing strong hydrogen bonding acceptors exhibited a better inhibitory effect on the crystal growth, in comparison with those containing strong bonding donors [25]. 

**Figure 3:**
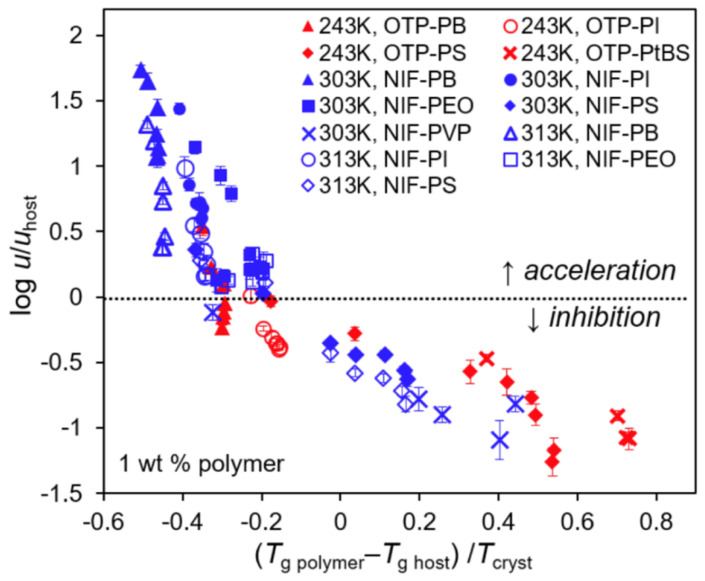
A master curve of polymer effects on crystal growth as a function of (*T*_g,polymer_ − *T*_g,host_) /*T*_cryst_). Here, the selected model compounds are o-terphenyl (OTP) and nifedipine (NIF). The selected polymers are polybutadiene (PB), polyisoprene (PI), poly(ethylene oxide) (PEO), polystyrene (PS), poly(*tert*-butylstyrene) (PtBS), and polyvinylpyrrolidone (PVP), with various molecular weights. Adapted from Ref. [14] with permission. Copyright © 2017 American Chemical Society.

The physical properties of a polymer have also been reported to strongly affect the crystal growth of amorphous pharmaceutical solids [14,16]. For instance, despite the constant drug–polymer hydrogen bonding interaction, the inhibitory effect of PVP on the crystal growth of felodipine can be enhanced with an increasing molecular weight of PVP [42]. In addition, the difference between the *T*_g_ of the drug and the *T*_g_ of the selected polymer was also proposed to be one important factor affecting the crystal growth [43]. For instance, a low concentration of biocompatible polymer polyhydroxybutylate can effectively inhibit the crystal growth of drugs with a low *T*_g_ [43]. For comparison, the same content of polyhydroxybutylate could act as a crystal growth accelerator for drugs with a much higher *T*_g_ [43]. A study by Powell et al. shows that the accelerating and inhibitory effects of polymers on the crystal growth of nifedipine below its *T*_g_ correlate well with the *T*_g_ of the polymer [43]. No strong correlations could be observed between the strength of the drug–polymer hydrogen bonding interactions and the crystal growth kinetics in these nifedipine–polymer binary systems [43]. Poly(ethylene oxide) (PEO), a low-*T*_g_ polymer, has been reported to effectively accelerate rather than inhibit the crystal growth of nifedipine [43]. Powell et al. propose that the mobility of the polymer chain instead of the strength of the drug–polymer hydrogen bonding interaction plays a controlling role for the crystal growth of amorphous drugs in these binary systems [16]. In a recent study, Huang et al. reported that the effect of the polymer on the crystal growth rate of nifedipine and *o*-terphenyl follows one master curve as a function of (*T*_g,polymer_ − *T*_g,host_)/*T*_cryst_), where *T*_cryst_ represents the temperature for crystal growth (Figure 3) [14]. They propose that the effect of the polymer on the crystal growth mainly depends on its segmental mobility relative to the mobility of the host molecule [14]. A local polymer-rich region is expected to be created at the crystal–liquid interface during the crystal growth process. Prior to entering the crystalline phase, host molecules must traverse this polymer-rich region at rates determined by the segmental mobility of the polymer. 

Understanding whether the effect of the polymer on the crystal growth of a polymorphic system exhibits strong polymorphic dependence is important, particularly for revealing the underlying mechanism of the role of polymer additives on crystallization. Kestur and Taylor compared the role of PVP on the crystal growth kinetics of two different polymorphs of felodipine [44]. The addition of PVP exhibits similar inhibitory effects on the crystal growth of both polymorphs, as evidenced by the similar ratios between the growth rate of pure felodipine and that of the system with PVP for these two polymorphs. It is proposed that the similar inhibitory effects of the polymer on drug polymorphs are mainly attributed to the effect of the polymer on the amorphous matrix, rather than the crystal surface. For comparison, Zhang et al. found that the crystal growth of form II of itraconazole is more sensitive to the inhibitory effect of PVPVA64 and hydroxypropylmethyl cellulose acetate succinate (HPMCAS), than of form I [45]. They proposed that this result mainly originates from the much stronger polymer adsorption on the crystal surface of form II, leading to a higher crystal–liquid interfacial free energy. Similar selective accelerating or inhibitory effects of polymers on the crystal growth of different polymorphs have also been reported in indomethacin solid dispersion with PVP or PEO, respectively [46,47]. Madejczyk et al. found that acetylated maltose can effectively inhibit the crystal growth of the α-form and β-form of nifedipine [48]. However, only the α-form of nifedipine exhibits an increase in the activation energy barrier of crystal growth in the presence of acetylated maltose [48]. 

**Figure 4:**
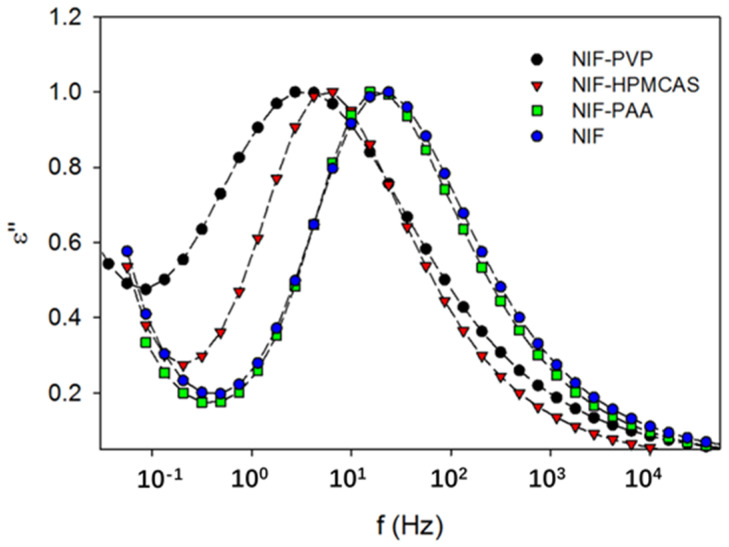
Dielectric loss peak of pure nifedipine and nifedipine systems with 10% PVP, HPMCAS, and PAA at 60 °C. Adapted from Ref. [49] with permission. Copyright © 2015 American Chemical Society.

One explanation for the effect of the polymer on crystallization is that the polymer can change the molecular mobility of the system [49,50]. Some studies also reveal that the molecular mobility can be determined to predict the crystallization in ASD, which would enable rational polymer selection [50,51]. Kothari et al. investigated the influence of drug–polymer hydrogen bonding interactions on crystallization kinetics and on molecular mobility in nifedipine solid dispersions [49]. The FT-IR spectrum revealed that the strength of the hydrogen bonding interactions is ranked in order as PVP > HPMCAS > poly(acrylic acid) (PAA) [49]. The dielectric loss peak of the PVP-doped nifedipine amorphous system appears at a much lower frequency in comparison with that of the HPMCAS-doped system, indicating that PVP imposes a more pronounced effect on decreasing the molecular mobility (Figure 4). For comparison, the dielectric loss peak of PAA-doped nifedipine virtually overlaps with that of pure nifedipine, suggesting that the effect of PAA on molecular mobility is negligible. The crystallization rate of nifedipine solid dispersion is also ranked in the order of PVP > HPMCAS > PAA. The authors propose that stronger hydrogen bonding interactions between the drug and the polymer can lead to longer relaxation times (lower molecular mobility); consequently, a higher resistance against drug crystallization. In a subsequent study, a linear relationship was identified between the polymer concentration and the structural relaxation time at a given temperature range [50]. With the polymer concentration increasing, the structural relaxation time becomes longer, reflecting a decrease in the global molecular mobility. A model can be built by using molecular mobility as an indicator for predicting the crystallization of the drug in solid dispersions. Mohapatra et al. investigated the effect of the molecular weight of PVP on the molecular mobility and crystallization of indomethacin in these solid dispersions [52]. With the increase in the molecular weight of PVP, longer α-relaxation times for indomethacin solid dispersion are observed, indicating a decrease in the molecular mobility. The inhibitory effect on the crystallization indomethacin is enhanced with the increase in the polymer molecular weight. Solid-state NMR reveals that the extent of drug–polymer hydrogen bonding interaction is independent of the polymer molecular weight [52]. Given the reasonably similar temperature dependence of molecular mobility and viscosity over the experimental polymer concentration, it is concluded that the effect of polymer molecular weight on the crystallization is mainly attributed to the different increases in the viscosity, decreasing the system’s molecular mobility [52]. 

Mistry et al. investigated the effects of different drug–polymer interactions on the molecular mobility of a weakly basic drug, ketoconazole, in polymer-based solid dispersions [53]. They found that ketoconazole can form an ionic interaction with poly(acrylic acid) (PAA), a hydrogen bonding interaction with poly(2-hydroxyethel methacrylate) (PHEMA), and weaker dipole–dipole interactions with PVP. Dielectric spectroscopy revealed that the global molecular mobility of ketoconazole solid dispersion is ranked in the order of PVP > PHEMA > PAA. Moreover, the formation of strong ionic interactions between ketoconazole and PAA leads to a dramatic and disproportionate decrease in global mobility with an increase in polymer concentration. More importantly, the decreased molecular mobility due to the strong molecular interactions further cause delays in crystallization [53]. In a subsequent study, isothermal crystallization experiments were conducted, and the crystallization rate constant was calculated by applying the modified Kolmogorov–Johnson–Mehl–Avrami (KJMA) model [51]. Herein, the decrease in the magnitude of the crystallization rate constant is strongly correlated with the formation of strong drug–polymer interactions. The coupling coefficient (~0.5), a measure of the extent of coupling between molecular mobility and crystallization kinetics, is determined to be ~0.5 in amorphous ketoconazole, with or without the presence of these polymers. The value of the coupling coefficient is unaffected by the presence of a low-concentration polymer and the strength of the molecular interactions between the drug and the polymer. On the basis of the relatively constant coupling coefficient (~0.5), one model is established, and it predicts that the crystallization times agree reasonably well with the experimental results. 

**Figure 5:**
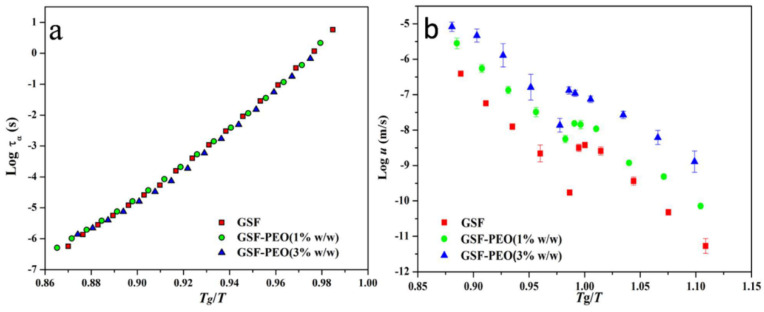
(**a**) Plots of α-relaxation time; (**b**) plots of crystal growth rates on a *T*_g_/*T* scale. Adapted from Ref. [54] with permission. Copyright © 2017 American Chemical Society.

**Figure 6:**
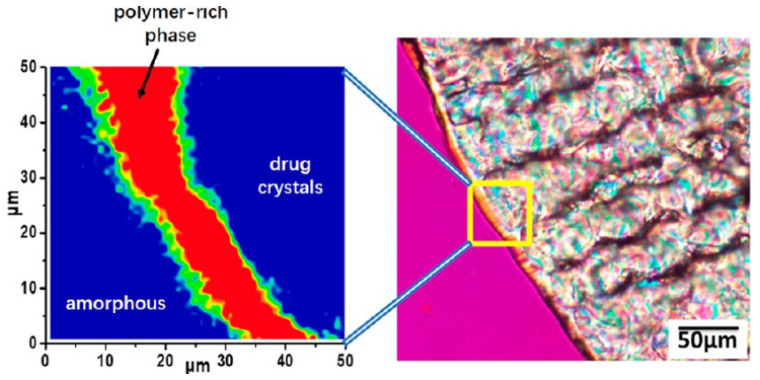
Raman mapping and photomicrograph of crystal growth front of GSF in griseofulvin–PEO binary system. Adapted from Ref. [55] with permission. Copyright © 2019 American Chemical Society.

Some argue that global molecular mobility is only partially responsible for affecting the crystallization, and that other factors should also be taken into consideration [46,54,55,56,57]. Shi et al. investigated the effect of low-concentration (1% and 3% *w*/*w*) poly(ethylene oxide) (PEO) on the global molecular mobility and crystal growth of griseofulvin [54]. The addition of 3% *w*/*w* PEO is observed to substantially accelerate the crystal growth of griseofulvin by nearly two orders of magnitude for both the glassy and supercooled liquid states. Liquid dynamics characterized using dielectric spectroscopy also revealed that the presence of PEO can effectively increase the global molecular mobility, as evidenced by a decrease in α-relaxation times [54]. From the perspective of liquid dynamics, the increase in the global molecular mobility is mainly attributed to the plasticization effects of the PEO additive, which is strongly supported by the overlapping of the α-relaxation time curves of griseofulvin with and without the presence of PEO on a *T*_g_/*T* scale (Figure 5a). However, on the same *T*_g_/*T* scale, the crystal growth rates of griseofulvin with PEO do not overlap with that of pure griseofulvin (Figure 5b). In addition to the increase in the global mobility, the accelerating effect of PEO on the crystal growth of griseofulvin is also strongly correlated with its high segmental mobility. A recent study with more drugs reveals that the accelerating effect of low-concentration PEO is independent of the *T*_gs_ of the drugs, the change of global molecular mobility, and the drug–polymer Flory–Huggins interactions [57]. Zhang et al. report that PEO can enrich at the crystal–liquid interface during the process of crystal growth of griseofulvin (Figure 6) [55]. Herein, the heterogeneous distribution of griseofulvin and PEO at the crystal growth front is investigated using confocal Raman microscopy, energy-dispersed X-ray spectroscopy, and scanning electron microscopy. The authors propose that the local enrichment of PEO at the crystal–liquid interface rather than the polymer concentration in the bulk predominantly controlled the crystal growth of griseofulvin. 

Polymer enrichment at the crystal–liquid interface can also explain the selective effects of polymer on the crystal growth of drug polymorphs [56,57]. For instance, 3% *w*/*w* PEO can significantly increase the crystal growth rates of the γ- and α-form of indomethacin [57]. For comparison, the same content of PEO exhibits a negligible effect on the crystal growth of the δ-form of indomethacin. In a subsequent study, it was found that PEO can be significantly enriched at the growth front of γ- and α-indomethacin, but not at that of δ-indomethacin [56]. It is proposed that the discrepant effects of PEO on the crystal growth of indomethacin polymorphs are mainly attributed to the different degrees of polymer adsorption on the crystal surfaces of these polymorphs. In addition, the reduction in the crystallization activation energy of indomethacin polymorphs in the presence of PEO also follows the same order as γ-form > α-form > δ-form. 

Recent studies show that tensile stress could induce extensive network fracture, facilitating heterogeneous nucleation and fast crystallization [58,59]. Su et al. reported a direct connection between fracture and crystal nucleation by performing an extensive statistical study [58]. Fast crystal growth along the cracks created via fracture is also reported in griseofulvin and o-Terphenyl glasses [59,60]. In addition, Powell et al. propose that the rapid glass-to-crystal (GC) growth is attributed to the fracture and surface mobility [59]. In the proposed model of GC growth, fracture steadily creates free surface and small voids, accelerating the local crystal growth by taking advantage of surface mobility. The addition of polymer has been demonstrated to effectively increase the fracture resistance of molecular glasses under tension [61]. The enhancement in the fracture resistance via the addition of polymer is mainly attributed to the increase in the fracture surface area as the tips of cracks circumvent the pervaded volume of encountered polymer chains. Moreover, the fracture resistance of molecular glasses can be enhanced by increasing the molecular weight of the polymer additives.

### 2.3. Effects of Polymers on Miscibility and Phase Separation

One important aspect in implementing the strategy for delivering poorly water-soluble drugs via polymer-based solid dispersions is to understand the solubility of drugs in polymers [62,63]. The solubility of a drug in a polymer, and defining the upper limit of drug loading with the tendency of crystallization, is relevant for the rational selection of a polymer for ASD. One method for calculating the solubility of a drug in a polymer is to compare the *T*_g_ of the pure polymer and the eventual *T*_g_ of systems by using the moisture to induce drug crystallization in the polymer mixture [64]. However, this method for measuring drug solubility in polymer is limited in the dry state by drawing water into this system. Marsac et al. developed predictive models for calculating the drug solubility in polymers on the basis of the Flory–Huggins theory of liquids [65,66]. Herein, the drug solubility in a polymer is estimated by using the calculated interaction parameter, which is strongly related to the depression in the melting point (*T*_m_) and the ideal entropy of mixing [65]. In addition, the compatibility and phase stability of the drug–polymer systems can also be predicted by estimating the free energy of mixing through the interaction parameters [66]. 

Tao et al. developed one thermodynamic method for measuring the solubility of a drug in a polymer by using DSC [67]. They proposed that cryogenic milling is an effective approach for achieving proper mixing, facilitating the determination of the equilibrium endpoint of the dissolution of drug molecules in a polymeric matrix. By applying this DSC method, the solubility of a small-molecule crystal is first measured near *T*_g_. The solubility at *T*_g_ can also be obtained via an extrapolation of the measured data to the lower temperature. On the basis of the above-mentioned scanning method, Sun et al. proposed a new annealing method for complementing the measurement of solubility of a drug in a polymer, using DSC [68]. Herein, a drug–polymer mixture prepared via cryo-milling is annealed at various temperatures and evaluated by whether undissolved crystals remain, thus obtaining the upper and lower bounds for the equilibrium solution temperature. The annealing method yields the same results as the scanning method at relatively high temperatures, while yielding slightly different results at lower temperatures. The solubility of the drug in the polymer is strongly dependent on both the drug and the polymer. In the case of nifedipine, the dissolving power of the polymer is ranked in the order as PVP K12 > PVPVA > PVAc. For the same polymer investigated, indomethacin exhibits a stronger dissolving power in comparison to nifedipine. In a recent study, Shi et al. compared the solubility of different drugs in PEO using the annealing method [57]. With an increase in the PEO content, the activities of three drugs decreased significantly and the magnitude of decrease followed the order of indomethacin > nifedipine > griseofulvin. Herein, the Flory–Huggins parameters of these systems are calculated as being −0.29, −1.22, and −2.76, respectively, indicating that the miscibility between the drug and the polymer is ranked as indomethacin > nifedipine > griseofulvin. These results are mainly attributed to the different functional groups of these drugs for determining the formation of hydrogen bonding interactions. 

Tian et al. proposed one small-scale method for predicting and comparing the solubilities and miscibilities of drugs in polymer-based ASD, in combination with the Flory–Huggins theory [69]. The temperature dependence of the Flory–Huggins parameter *χ* of felodipine and the selected polymer (HPMCAS-HF and Soluplus) is calculated from the melting depression of the crystalline drug. A change in the melting peak is generally proposed to be strongly related to the formation of a new solid form, intermolecular interactions, molecular structure, molecular symmetry, the dissolving of the crystalline drug in polymer, etc. [70,71]. Herein, the values of *χ* calculated using the melting point depression method are comparable to those calculated by using the van Krevelen solubility parameter method. Phase diagrams of drug composition–temperature and drug–polymer mixing free energy are also constructed, and might be used for predicting the maximum drug solubility and amorphous drug miscibility. In addition, the phase separation of drug composition–temperature can also facilitate the identification of the temperature/ drug loading space for formulating robust solid dispersions. Knopp et al. compared the different methods for predicting drug–polymer solubility by using the Flory–Huggins theory [72]. They found that the results predicted using the recrystallization and melting point depression methods are similar. For comparison, the prediction using the dissolution endpoint method is consistently lower. In addition, compared with the dissolution endpoint and melting point depression methods, the recrystallization method exhibits a smaller confidence interval of prediction because of a better fit of the data to the model obtained from the Flory–Huggins theory. The Flory–Huggins interaction parameter *χ* is generally considered to be inversely proportional to the *T*_m_ of the drug in a polymer-based binary system. A highly sensitive DSC technique is used to detect the remaining residual crystalline drug at a temperature near to the estimated solubility curve, and to verify the proposed linear relationship [73]. It is found that this proposed linear relationship does not apply for compositions with a low drug content (<10 wt%), indicating that the Flory–Huggins interaction parameter depends on both the temperature and the composition. Recent studies also revealed that the thermodynamic modeling of drug–polymer can provide an informative framework for the design of robust ASD [74]. It is generally accepted that hot-melt extrusion is a well-developed industrially feasible technique for continuous, one-step, and solvent-free preparations of high-quality ASD and cocrystal [75]. A one-step continuous hot-melt extrusion facilitates the preparation of high drug loading ASD with enhanced physical stability [74]. Herein, the Flory–Huggins thermodynamic model gives a well-defined space for the interpretation and evaluation of the hot-melt extrusions, which are relevant for maintaining the quality of ASD [74].

Hydrogen bonding is proposed to be an important factor for determining the miscibility and supersaturation potential. However, the Flory–Huggins theory was never intended to be used in systems containing hydrogen bonding interactions [76]. Anderson and coworkers developed a molecular dynamic simulation for characterizing molecular interactions in a solid dispersion, and explored their effects on miscibility [77,78]. In the case of the indomethacin–PVP system, the solubility parameters of an amorphous drug and polymer are calculated to be 25.5 MPa^1/2^ and 19.0 MPa^1/2^, respectively. A small positive free energy of mixing is expected if the difference in the solubility between the drug and polymer is smaller than 7 MPa^1/2^. Compared to the pure amorphous indomethacin system, a molecular dynamics simulation reveals that the hydrogen bonds formed between indomethacin molecules in an indomethacin–PVP mixture are much fewer [77]. Herein, the fraction of indomethacin molecules involved in hydrogen bonds with other indomethacin molecules, which indicates the potential towards crystallization, is also vastly reduced. The loss of the hydrogen bonds between indomethacin molecules in the PVP-doped binary system is largely compensated for by the newly formed drug–polymer hydrogen bonds. If the molecular interactions between drug and polymer are taken into account, the Flory–Huggins interaction parameter is calculated to be −0.61 ± 0.25. This result indicates complete miscibility in the indomethacin–PVP binary system, which is in agreement with direct experimental observations. The miscibility of HPMC with a hydrophobic drug felodipine is also investigated, using both the solubility parameter method and the Flory–Huggins theory [78]. The difference in the calculated solubility parameters of the drug and polymer is 2.8 MPa^1/2^, and it is much smaller than 7 MPa^1/2^, which defines the miscibility criterion. Complete miscibility for the felodipine system vs. HPMC compositions is predicted using the drug–polymer Flory–Huggins interaction parameter (−0.20 ± 0.07), in agreement with experiments. However, the increase in water content can disrupt the drug–polymer hydrogen bonds favoring miscibility. A molecular dynamics simulation is used for constructing a molecular structure for amorphous dispersions of ibuprofen alone, or with one of four polymers, including PVP, PVPVA, PVAc, and polystyrene (PS) [79]. Distributions of hydrogen bonds and contributions from internal, electrostatic, and van der Waals interactions to miscibility and mobility are investigated as a function of drug concentration. Drug–polymer miscibility is accessed by determining the concentration-dependent Flory–Huggins parameters. The values of the Flory–Huggins parameter of ibuprofen and PVP are −0.9 to −1.8, with a plateau of nearly 50% *w*/*w* PVP, while the value of the ibuprofen–polystyrene Flory–Huggins parameter fluctuates near zero, indicating that ibuprofen is more soluble in PVP than in polystyrene. The Flory–Huggins parameter of ibuprofen in the polymer varies in the compositions of pyrrolidone and acetate, and the miscibility is ranked in the order of ibuprofen–PVP > ibuprofen–PVPVA > ibuprofen–PVA. In addition, with an increase in the content of PVP, the local mobility of ibuprofen measured using atomic fluctuation decreases, which is attributed to the increase in drug–polymer hydrogen bonds. However, atomic fluctuations reveal an opposite trend for the local mobility of ibuprofen and the content of polymer in the ibuprofen–polystyrene system [76]. This unexpected effect mainly arises from the disruption of hydrogen bonds on dilution [79]. 

Incorporating surfactants into ASD can facilitate the dissolution of the drug, while also strongly affecting the miscibility [80]. Gumaste et al. systemically investigated the polymer–surfactant and polymer–drug–surfactant miscibility by using a film casting method. Herein, poloxamer 188, a crystalline solid surfactant, was used as the model surfactant. Soluplus^®^ and HPMCAS, two commonly used amorphous polymeric carriers, were chosen as the model polymers. It was found that the miscibility of poloxamer 188 in Soluplus^®^ is lower than 10% *w*/*w*, while its miscibility in HPMCAS is ≥30% *w*/*w*. In this drug–polymer–surfactant ternary system, the presence of poloxamer can drastically reduce the miscibility of itraconazole in Soluplus^®^, from 40% *w*/*w* to lower than 10% *w*/*w*. For comparison, the addition of poloxamer shows a minimal effect on the miscibility of itraconazole in HPMCAS. It is also proposed that the poloxamer–HPMCAS mixture can be an ideal carrier for delivering poorly water-soluble drugs, from the perspective of both physical stability and drug release. 

Miscibility has been demonstrated to be an important characteristic of ASD that strongly affects the physical stability [62,81,82,83]. Tian et al. evaluated drug–polymer miscibility by using one fluorescence-based technique, and explored its correlation with the physical stability of ASD [81]. Herein, indomethacin was selected as the model drug, while HPMC, HPMCAS, and PVP are used as the model polymer. It was found that drug–polymer miscibility is greatly dependent on the selected polymer. The indomethacin–HPMCAS system exhibits minimal miscibility and is only miscible at relatively low drug loading in this system. The miscibility of indomethacin in HPMC and PVP is much higher in comparison with the indomethacin–HPMCAS system. The largest miscibility range is observed in indomethacin when formulated with PVP. A good correlation can be established between the phase separation and crystallization of indomethacin in these solid dispersions, indicating that miscibility can strongly affect the physical stability. In a very recent study, Sharma et al. investigated the relationships between molecular relaxation, quantitative drug–polymer miscibility, and phase separation in dipyridamole ASDs [82]. The miscibility of dipyridamole systems doped with co-povidone (CP), HPMCP, and HPMCAS was predicted by using the melting point depression method. A modulated DSC was performed to obtain the stretched relaxation time (*τ*_β_), and the experiment revealed that *τ*_β_ follows the order of dipyridamole–CP > dipyridamole–HPMCP > dipyridamole–HPMCAS ASDs. For the dipyridamole–CP system, modulated DSC revealed that the aged sample exhibits the single-phase behavior. For comparison, amorphous–amorphous and amorphous–crystalline phase separation can be observed in the aged dipyridamole–HPMCP and dipyridamole–HPMCAS ASDs, respectively, via modulated DSC. This phase separation phenomenon is further characterized via confocal laser scanning microscopy and X-ray micro computed tomography. It is proposed that good miscibility between the drug and polymer can be translated into a reduction in molecular mobility and superior physical stability. 

Iemtser et al. evaluated and compared the performance of the predictive model concerning the solid–liquid equilibrium curve and *T*_g_ line modeling for the solubility of ibuprofen in HPMC and HPMCAS [84]. The solubility of ibuprofen in these polymer matrices predicted from Perturbed-Chain Statistical Associating Fluid Theory (PC-SAFT) is much higher than those predicted using the empirical analytical approach. A greater thermodynamic stability and higher resistance to crystallization is expected from PC-SAFT during storage. For these predictive models, the obtained solubility of ibuprofen strongly depends on the applied parametrization strategy and the chosen extrapolation length at lower temperatures. As predicted via PC-SAFT, the ibuprofen–polymer system containing a high drug concentration would split into two liquid phases. However, the prediction is in contradiction to the results from the experiment. Moreover, it is also proposed that an overestimation of stability is expected from the Gordon–Taylor equation, due to its inadequate consideration of the measured *T*_g_. Bochmann et al. compiled experimental data, measurement techniques, and predictive models for the solubility of API in polymorphic matrices [85]. Herein, the solubility results included 37 APIs, two sugar derivatives, and seven polymers widely used in the preparation of ASD via spray dying and hot-melt extrusion. The prediction models mainly included melting point (*T*_m_) depression, the measurement of the dissolution endpoint, indirect determination via *T*_g_, and the use of low molecular weight analogues. It was found that the solubility of API depends more on the specific analytical method than on the measurement technique. Herein, no simple relationship can be observed between the solubility of API and the molecular weight of PVP. Among the polymers tested, API exhibited the highest solubility in PVP, while the lowest solubility was in polyvinyl acetate (PVAc). In the case of copovidone (COP), a copolymer consisting of PVP and PVAc, the API solubility changed with the ratio between PVP and PVAc. In addition, the descriptors of molecules related to API solubility were identified via a statistical assessment using recursive feature elimination. The eight identified descriptors related to API solubility were the number of donors of hydrogen bonding. Three descriptors were connected to *T*_g_, the hydrophobicity of API, the out-of-plane potential energy, the fractional negative polar van der Waals surface area, and the fraction in rotatable bonds.

Huang and Dai proposed that a drug/polymer binary solid dispersion can possibly form three different structures, largely depending on the drug–polymer compositions and the processing history [86]. The drug can be molecularly dispersed into the polymer and form a thermodynamically stable system if the drug loading is lower than its equilibrium solubility in this polymer. However, this desirable structure of the drug–polymer system only occurs at high temperatures or in the case of very low drug loading. If the drug and polymer are viewed as the solute and solvent, with the temperature decreasing, the drug–polymer system would form a supersaturated solution and favor the precipitation of the drug (solute) from the polymer matrix (solvent). As a result, crystalline drug particles are gradually produced in the polymer matrix. For comparison, a meta-stable structure can be formed if the drug crystallization has a much lower rate and a higher energy barrier than the amorphous phase separation. Herein, the drug can aggregate in the amorphous form and be dispersed into the polymer matrix. This phenomenon, termed amorphous–amorphous phase separation (AAPS), can occur very rapidly, particularly for the binary system entering the spinodal zone in which the occurrence of phase separation requires no free energy barrier [87]. 

**Figure 7:**
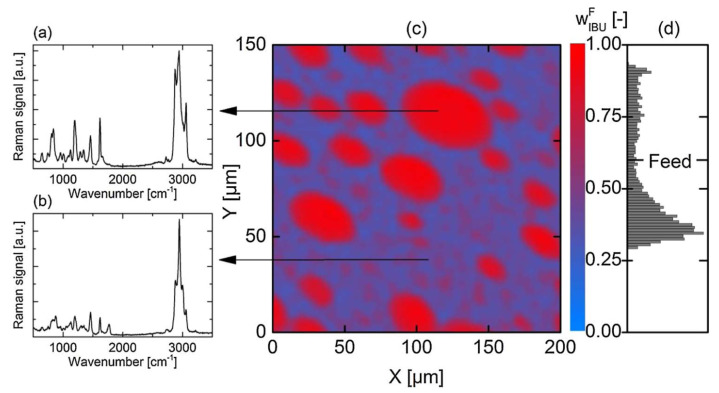
Raman mapping of ibuprofen–poly(lactic-co-glycolic acid) system containing 60 wt% ibuprofen. Raman spectra of drug-rich (**a**) and polymer-rich (**b**) regions. The obtained Raman map (**c**) and ibuprofen distribution from this Raman map (**d**). Adapted from Ref. [92] with permission. Copyright © 2018 Elsevier.

AAPS generally leads to a binary system consisting of drug-rich and polymer-rich phases [87,88]. As a result, crystallization can be enhanced in the drug-rich phase, due to the decreased polymer concentration in these regions. Purohit et al. investigated the miscibility of itraconazole and hydroxypropyl methylcellulose (HPMC) in the binary system, and observed amorphous–amorphous phase separation in the samples prepared using solvent evaporation [87]. Discrete itraconazole-rich domains are formed and discretely dispersed in the continuous HPMC phase. However, the DSC results showed one single *T*_g_, indicating the good miscibility of the drug and polymer in this binary system. It should be noted that *T*_g_ sometimes cannot be a reliable indicator of miscibility. A phase-separated system can sometimes exhibit one single *T*_g_, while a miscible system can sometimes have two *T*_g_s [89,90]. Li and Taylor investigated the miscibility behavior of telaprevir in a polymer-based solid dispersion as a function of polymer type and content, using atomic force microscopy coupled with nano-IR, nanothermal analysis, and Lorentz contact resonance measurement [91]. Phase separation could be observed in HPMC- and PVPVA-based solid dispersion at a telaprevir loading of above 10%. For comparison, phase separation in a HPMCAS-based system was observed at a drug loading of higher than 30–40%. Herein, the telaprevir-rich phase could form discrete domains with a size ranging from tens to hundreds of nanometers. Luebbert et al. report that the kinetics of amorphous–amorphous phase separation and the compositions of these phases can be quantitatively investigated using confocal Raman spectroscopy [92]. Amorphous–amorphous phase separation can occur in dry formulations with high drug loading. In the case of an ibuprofen–poly(lactic-co-glycolic acid) system containing 60 wt% drug, droplet-shaped drug-rich domains can be clearly observed in the polymer-rich surrounding matrix via Raman mapping at 40 °C (Figure 7). The droplet size of the drug-rich phase is measured to be in the range of 5–30 μm. The Raman spectrum of the drug-rich droplet shown in Figure 7a is similar to that of the pure drug system, and the concentration of ibuprofen is determined to be 92.3 wt%. The spectrum of the polymer-rich matrix is used to determine the lower concentration of ibuprofen of 35.4 wt%, in comparison with the drug-rich phase [92]. The concentration of ibuprofen in the experimentally measured formulations vary from 28.7 to 93.4 wt% (Figure 7d). It should be noted that only 1.22% of all spectra exhibit the same concentrations as the homogeneous system containing 60 wt% ibuprofen. 

In a very recent study, Yang et al. investigated the effects of four various surfactants on water-induced phase separation in ritonavir–PVPVA ASDs [93]. The kinetics and morphology of phase separation induced via exposure to high humidity were monitored using fluorescence confocal microscopy. The compositions of the phase-separated domains and surfactant distribution were characterized using optical photothermal IR analysis. ASDs without the presence of a surfactant exhibited a lacey or bicontinuous morphology. For comparison, ASDs containing Tween 80 and SDS exhibited discrete circular drug-rich domains. Continuous and discrete drug-rich domains were both observed in ASDs containing Span 20. However, the addition of Span 80 only led to the formation of a continuous drug-rich phase. Surfactant distribution analysis showed that different surfactants exhibit different affinities for drug-rich or polymer-rich phases. SDS, the most hydrophilic one among these four surfactants, is found to mainly reside in the polymer-rich region. For comparison, surfactants exhibiting high hydrophobicities prefer to distribute in the drug-rich region. 

## 3. Role of the Polymer on Dissolution and Supersaturation

In general, the enhanced in vitro performance of ASD is mainly attributed to the so-called “spring and parachute” effect [4,86]. Herein, “spring” represents the fast dissolution that creates the supersaturated drug concentration in the solution. “Parachute” represents the maintenance of supersaturation, for enhanced absorption and superior bioavailability. A rapid crystallization of the amorphous drug in solution would cause a failure of the “spring and parachute” effect for enhancing the bioavailability. 

In our previous publication, some key findings are summarized for the studies regarding the dissolution and supersaturation of ASD over the past decades [4]. In addition to the physicochemical properties of a drug and the formulation design, polymer selection is also one of the most important factors affecting the supersaturation of ASD. Konno et al. investigated the effect of different polymers on the dissolution behavior of felodipine ASD [94]. The felodipine concentration versus time profiles were plotted for a supersaturated solution in a pure amorphous system and ASD. Herein, HPMCAS-doped ASD exhibited the highest level of drug supersaturation with the greatest length of time, which is mainly attributed to its best effect on inhibiting crystal growth. For comparison, PVP exhibits the least inhibitory effect on drug crystal growth. Studies with several polymers, including some novel cellulose derivatives by Ilevbare et al., found that the inhibitory effect of the polymer on drug crystallization in solution was strongly related to the hydrophobicity, amphiphilicity, and semi-rigid structure of the polymer [95,96]. 

The drug–polymer ratio is also proposed to be another key factor strongly affecting the dissolution behavior of drugs in ASD [97,98,99,100]. Saboo et al. comprehensively evaluated the release mechanism of felodipine ASD containing various polymers as a function of drug loading [99]. Herein, the selected polymer featured different hydrophobicities. In the case of relatively hydrophilic polymers, including PVP, PVPVA, and HPMC, drug release in the low drug loading ASD was polymer-controlled. For comparison, drug release in ASDs containing higher drug loading exhibited a similar behavior to the pure amorphous drug system. In the case of more hydrophobic polymers, HPMCAS and Eudragit^®^S100, both the drug and polymer in ASD are polymer-controlled for drug loading as high as 50%. With the drug loading further increasing, drug release exhibits a more gradual decline. It is summarized that a drug in ASD containing a hydrophilic polymer releases more rapidly in comparison with those containing a hydrophobic polymer in the polymer-controlled dissolution region. ASDs containing a hydrophilic polymer are advantageous in drug release for low drug loading systems. A “trade-off” is proposed to be taken into consideration for polymer selection in preparing ASDs. Herein, ASDs containing more hydrophilic polymer at low drug loading support faster release rates. For comparison, high drug loading ASDs containing more hydrophobic polymer are compromised by the reduced drug release rates. 

It is proposed that formation of nonspecific drug–polymer hydrophobic interactions is a key factor determining the effects of the polymer on the crystal growth of a drug in solution [95]. In the case of celecoxib and efavirenz, specific intermolecular interactions between these drugs and PVPVA, a hydrophilic polymer, are also important for the inhibitory effect on drug crystal growth [95]. These specific and nonspecific drug–polymer interactions are most likely to facilitate polymer adsorption onto the surface of the crystalline drug, thus affecting the drug’s crystal growth. In addition, the effect of the polymer on drug crystallization also strongly depends on the crystal growth rate of a drug. Recent studies report a new high-throughput controlled polymerization method for designing customized polymer additives as a precipitation inhibitor for the recrystallization of an amorphous drug [101,102]. These molecularly customized excipients provide different drug–polymer noncovalent interactions, and thus result in the discrepancy in maintaining supersaturation in solution [101].

Some drug–polymer molecular interactions that stabilize ASDs in the solid state are also proposed to be important and relevant in maintaining supersaturation in solution [103]. The formation of ionic interactions between ketoconazole and poly(acrylic acid) leads to a reduction in global molecular mobility and the enhanced physical stability of ASDs in the solid state. A study on in vitro dissolution shows that ketoconazole-poly(acrylic acid) ASDs containing a low content of polymer (4–20% *w*/*w*) can effectively maintain drug supersaturation for a long duration. In addition, X-ray diffractometry reveals the ability of these ASDs in resisting drug crystallization in solution. The solution-state drug–polymer interactions accompanied by reduced drug diffusivity are detected in ketoconazole-poly(acrylic acid) ASDs, which are mainly responsible for the resistance to drug crystallization and prolonged supersaturation. 

Chen et al. investigated various physiochemical properties and processes related to drug–polymer–water interactions in various ASDs of three different drugs in PVPVA or HPMCAS, and correlated these with the dissolution performance of ASDs [104]. Herein, these characterized physiochemical properties and processes included the tendency for drug crystallization in an aqueous solution, changes of drug–polymer interactions upon moisture exposure, supersaturation of a drug with a polymer, the dissolution kinetics of a polymer, etc. Ketoconazole–HPMCAS ASD has been demonstrated to outperform all other studied systems in various dissolution conditions. This superior dissolution performance is a result of the joint action of multiple factors, including the low crystallization tendency of ketoconazole, strong ketoconazole–HPMCAS interactions, and its robustness against water disruption, dissolution, and the ability for HPMCAS to maintain supersaturation. The authors propose that the only feasible option for a rapidly crystallized drug in the absence of strong drug–polymer interaction is to reduce the drug loading in ASDs. In addition, an ASD/water Flory–Huggins parameter plot is constructed to reveal the physical stability of the drug–polymer interaction. Two quantitative parameters, the “supersaturation parameter” and the “dissolution performance parameter”, are also defined and are verified as being highly valuable for comparing different drug–polymer ASDs. 

**Figure 8:**
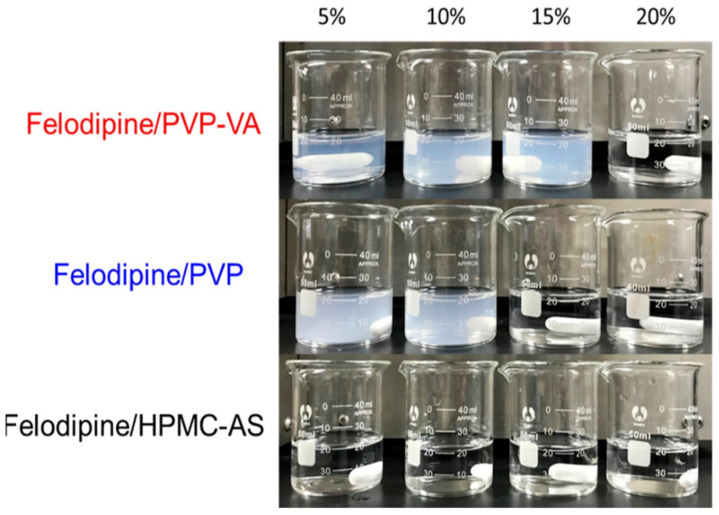
Appearance of dissolution media after 5 min of dissolution of Felodipine containing PVPVA, PVP, or HPMCAS in 0.05 M hydrochloric acid solution as a function of drug loading. Adapted from Ref. [105] with Copyright © 2022 American Chemical Society.

In a very recent study, Liu et al. investigated the effects of drug–polymer interactions on the dissolution performance of felodipine ASDs containing three different polymers as a function of drug loading [105]. Felodipine–PVPVA ASDs containing a low drug loading (<15%) exhibit rapid dissolution with the generation of nano-species in 0.05 M hydrochloric acid solution (Figure 8). Under the same dissolution conditions, rapid dissolution and nano-species generation can only be observed in felodipine-PVP ASDs containing <10% drug loading. For comparison, felodipine–HPMCAS ASDs always exhibit slow drug release without the generation of nano-species. In pH 6.5 phosphate-buffered saline (PBS), rapid dissolution with nano-species generation occurs in felodipine–PVPVA ASDs containing less than 10% drug loading. In the case of felodipine–PVP systems, a similar phenomenon occurs only in the ASDs when the drug loading is 5%. However, rapid dissolution accompanied with the generation of nano-species can be observed in felodipine-HPMCAS ASDs with 20% drug loading in PBS (pH 6.5). Flory–Huggins interaction parameters reveal that the strongest attractive drug–polymer interactions are in felodipine–PVPVA ASDs, followed by felodipine–PVP ASDs. For comparison, the positive Flory–Huggins parameter in felodipine–HPMCAS ASDs suggests that no attractive drug–polymer molecular interactions are present. In the case of felodipine ASDs containing PVPVA or PVP, drug–polymer molecular interactions are also found to be resistant to water, as evidenced by the results of dynamic vapor sorption. These water-resistant drug–polymer interactions are proposed to be responsible for nano-species generation, facilitating rapid drug dissolution at the initial stage. For comparison, the drug–polymer interactions in felodipine–HPMCAS ASDs vary significantly with the change in pH, due to the pH dependency of this polymer. 

The inhibitory effect of the polymer on drug crystallization in solution can also be affected by several other factors, including rigidity, conformation, aggregation state, and the substitute type of the polymer [106,107,108,109]. Schram et al. investigated the effect of the conformation of HPMCAS adsorbed on the drug crystal surface on the inhibitory effect of the polymer on the crystal growth of felodipine [108]. HPMCAS exhibits different conformations on the drug crystal surface at pH 3 and pH 6.8, due to its various ionization states. Atomic force microscopy reveals that HPMCAS uniformly adsorbs on the crystal surface at pH 6.8. For comparison, HPMCAS adsorbs less uniformly and forms coiled globules on the surface at pH 3. The conformation of HPMCAS at pH 3 is mainly responsible for its reduced effectiveness on the crystal growth of felodipine. It is expected that globule formation leaves more growth sites for drug molecules to attach on the crystal surface, thus rendering the inhibitory effect of the polymer. Wang et al. proposed that the key mechanisms of drug supersaturation and the crystallization inhibition of HPMCAS are the aggregation behaviors of the polymer and the drug–polymer affinity [109]. Using a higher polymer grade or a lower pH condition could lead to the higher aggregation of HPMCAS, as measured using a combination of static and dynamic light scattering. The increase in aggregation number correlates well with the enhanced drug supersaturation and the inhibitory effect on crystallization. Moreover, the amount of polymer that co-precipitates with the drug, an indicator of drug–polymer affinity, also exhibits a positive correlation with enhanced crystallization inhibition and prolonged drug supersaturation. 

It might not be always possible to prepare a completely amorphous solid dispersion. Crystals generated in various environments would exhibit different properties, which might affect crystallization in solution and the dissolution of ASD. Que et al. used a needle-like crystallizer, paclitaxel, as a model drug to explore the effects of crystal seeds on the dissolution of ASD [110]. Paclitaxel crystal seeds formed under various conditions exhibit different size distributions, interface structures, and different available growth areas. These different properties of seeds lead to variations in growth rates, and thus affect the supersaturation profile. For instance, it has been observed that paclitaxel seeds with a low aspect ratio exhibit more significant effects on the de-supersaturation rate, in comparison with those with a higher aspect ratio. In addition, the reduction in the crystal growth rate of paclitaxel in the presence of a polymer is mainly attributed to polymer adsorption on the crystal surface of drug. It is proposed that the available growth area and the interface structure, rather than the mass of crystal seeds, is relevant to the dissolution performance of ASD.

In the case of ASD, one interesting phenomenon, termed as liquid–liquid (or glass–liquid) phase separation (LLPS or GLPS), occurs once the free drug in the solution exceeds the amorphous solubility without crystallization [4,111]. Herein, the amorphous solubility in solution represents the theoretical maximum concentration of the molecularly dissolved drug. The occurrence of LLPS would lead to the formation and simultaneous existence of colloidal drug-rich and drug-poor phases in the solution. These dispersed and colloidal nano-droplets of the drug-rich phase are generated by the precipitation of excessive drug above the concentration of amorphous solubility. For a comparison, the drug-poor phase, also named as the water-rich phase, exhibits a drug concentration equivalent to amorphous solubility. This feature leads to one important application of LLPS for measuring the amorphous solubility of a drug in solution. Moreover, the drug concentration at which ASD undergoes LLPS is also closely related to the flux in passive membrane transport, one process that is important for oral bioavailability. 

Sugihara et al. investigated phase behaviors and the supersaturation profiles of pazopanib hydrochloride at initially low and then rapidly increased pHs [107]. The supersaturation degree of pazopanib is enhanced by approximately 600-fold at pH 6.5 in the presence of HPMC, a crystallization inhibitor. The maximum free drug concentration in solution for the ASD containing clinical dose is dictated by amorphous solubility. For comparison, systems that exceeded the amorphous solubility with an increase in pH will undergo LLPS and generate amorphous colloidal drug-rich particles. It is also observed that the presence of HPMC could delay the appearance of free base crystals of pazopanib. Herein, these behaviors in the pH changing experiments of pazopanib hydrochloride containing various drug doses can be explained by crystallization kinetics in solution and the pH-solubility phase diagram. 

In addition to the physicochemical properties of the drug and liquid media, polymer type, content, and the addition of surfactants can affect the formation and duration of LLPS. For instance, Ueda et al. investigated the different polymer substituent types (LF grade and HF grade) of HPMCAS on the amorphous solubility and membrane transport of drugs [106]. HPMCAS-HF exhibits a higher extent of acetate and a lower extent of succinate substituents, in comparison with HPMCAS-LF [106]. The amorphous solubility of the drug could be substantially reduced by HPMCAS-HF, while a minor effect was observed in the presence of HPMCAS-LF. ^1^H nuclear magnetic resonance (NMR) spectroscopy reveals that HPMCAS-HF can be extensively distributed into the drug-rich phase generated by LLPS, a result of the higher hydrophobicity of HPMCAS-HF. A reduction in the amorphous solubility of the drug by HPMCAS-HF is mainly attributed to the polymer distribution into the drug-rich phase. In addition, a decrease in the amorphous solubility of the drug leads to a reduction in drug transport in absorptive dissolution testing. 

Saboo et al. reported a change in the dissolution behavior of nilvadipine– and cilnidipine–PVPVA ASDS at a relatively low drug loading (<20%) [112]. The dissolution of these ASDs is found to switch from the rapid, simultaneous release of the drug and polymer to incongruent release and slow drug release. One interesting finding is that only ASDs exhibiting congruent release would undergo LLPS. As a result, amorphous drug-rich aggregates with ~300 nm size are generated. For ASD tablets showing incongruent drug release, a characteristic “pit” could be identified on the tablet surface upon dissolution. This phenomenon is mainly attributed to the faster polymer release than drug release, as evidenced by the drug-rich composition around these “pits”. A competition between matrix phase separation and drug release is proposed to explain the switch between the congruent and incongruent release of drug and polymer. Herein, drug release is much faster than matrix phase separation in the ASDs with a low drug loading. For comparison, matrix phase separation driven by water absorption occurs much more rapidly in the ASDs containing a high drug loading. 

The addition of a surfactant can strongly affect the inhibitory effect on drug crystallization and the maintenance of supersaturation [113]. In the case of posaconazole–HPMCAS ASDs, pre-dissolved HPMCAS in solution is demonstrated to be unable to delay the LLPS of posaconazole. Posaconazole-rich amorphous precipitates are formed, and the content of the polymer in the precipitates was ~16–18%. The co-precipitated HPMCAS is found to substantially delay drug crystallization in the posaconazole-rich phase to more than 4 h. For comparison, a fast crystallization tendency is observed in pure amorphous posaconazole in solution, as evidenced by the crystallization peak detected within 10 min. The co-existence of sodium lauryl sulphate (SLS) is revealed to negate the inhibitory effects of HPMCAS on drug crystallization in posaconazole-rich amorphous precipitates. As a result, the fast crystallization of posaconazole can be observed within 30 min. These results are mainly attributed to the assembly of SLS around HPMCAS, which could competitively interact with HPMCAS. These findings correlate well with the in vivo performance of posaconazole–HPMCAS ASD, with or without the presence of SLS. ASD containing SLS exhibits only 30% in vivo bioavailability as that of ASD without the presence of SLS. 

Ueda et al. investigated the effects of the polymer and three different surfactants on the size and stability of the colloidal ketoprofen-rich phase formed by LLPS [114]. Dynamic light scattering measurements revealed that the coarsening of the drug-rich phase cannot be effectively inhibited by only using the surfactant. For comparison, ketoprofen-rich droplet sizes can be maintained at ~200 nm via the combined use of the surfactant and HPMC, and the droplet size is much smaller than those of ASDs only containing HPMC. A decrease in the maximum achievable aqueous drug concentration is observed in the presence of HPMC, which is strongly related to the reduced thermodynamic activity due to the distribution of HPMC in the drug-rich phase. Herein, a reduction in the drug thermodynamic activity could translate to a decrease in supersaturation, leading to the reduced membrane flux. For the three surfactants, CTAB and Tween can extensively distribute into the drug-rich phase, while most of the SDS remains in the aqueous phase. Compared to the ASDs containing only HPMC, the combined use of HPMC with CTAB or Tween leads to a further decrease in the thermodynamic activity of the drug. These results originate from the mixing of both HPMC and these two surfactants with the drug-rich phase. For comparison, due to the limited amount of SDS distributing into the drug-rich phase, the addition of SDS leads to negligible effects on reducing the drug thermodynamic activity. 

It is widely accepted that amorphous solubility of a drug in solution can be affected by the presence of additional components once mixing with the drug-rich phase occurs [3]. For instance, an amorphous solubility of felodipine would change from ~10 to 177 μg/mL in the presence of Vitamin E TPGS micelles [115]. This increase in solubility is mainly attributed to the association of amorphous felodipine with the micelles. In the ASD containing two small molecular drugs, ritonavir and atazanavir, the maximum achievable supersaturation of one component decreases linearly with the increasing molar fraction of the other component during non-sink dissolution [116]. These results mainly originate from the decrease in the concentration at which drug-rich aggregates can form as the other component exists. The maximum achievable drug concentration in solution can also be altered by the presence of the polymer, particularly for poorly water-soluble polymers [117]. Herein, it is proposed that the achieved supersaturation mainly depend on the chemical potential of the drug in the drug-rich phase. Li et al. investigated the effects of various poorly water-soluble polymers on the maximum achievable concentration of lopinavir ASDs [117]. They found that the maximum achievable concentration of lopinavir is closely related to the drug–polymer interactions, as well as drug loading in the ASDs. Herein, the supersaturation of lopinavir can be vastly reduced by drug–polymer molecular mixing, due to the strong molecular interactions. For comparison, a drug–polymer phase separation is expected in the ASDs that show weak drug–polymer interactions. As a result, the supersaturation of lopinavir in ASDs is comparable to that of the pure amorphous drug system.

## 4. Conclusions

In conclusion, this review provides detailed descriptions and examples regarding the role of the polymer in physical stability and the in vitro performance of ASDs. We also discuss the rationality behind the polymer selection of ASDs. However, it should be noted that considerable challenges are still present for developing robust ASDs. One of the important issues is to understand polymer selection and functions in the different preparation techniques of amorphous solid dispersions, particular for newly emerged techniques such as microwave irradiation, 3D printing, supercritical anti-solvent processing, etc. Future work is warranted for the qualitative and quantitative assessment of the effects of the key properties of the polymer on nucleation, crystal growth, miscibility, phase separation, dissolution, and supersaturation; and for understanding the mechanisms on physical stability and the in vitro performance of ASDs. More systematic studies are required for elucidating the identifiable properties of polymers that affect the in vivo performance of ASDs, which also facilitates an in-depth understanding of the in vitro–in vivo performance of ASDs. Considerable attention should also be given toward understanding the role of polymers affecting the adsorption, metabolism, distribution, and excretion of ASDs. A new methodology also needs to be developed for revealing the exact correlations between the polymer properties and their effects on physical stability, and in vitro and in vivo performance.

## References

[1] Yu L. (2001). Amorphous pharmaceutical solids: Preparation, characterization and stabilization. Adv. Drug Deliv. Rev..

[2] Shi Q., Moinuddin S.M., Cai T. (2019). Advances in coamorphous drug delivery systems. Acta Pharm. Sin. B.

[3] Taylor L.S., Zhang G.G. (2016). Physical chemistry of supersaturated solutions and implications for oral absorption. Adv. Drug Deliv. Rev..

[4] Shi Q., Li F., Yeh S., Moinuddin S.M., Xin J., Xu J., Chen H., Ling B. (2021). Recent Advances in Enhancement of Dissolution and Supersaturation of Poorly Water-Soluble Drug in Amorphous Pharmaceutical Solids: A Review. AAPS PharmSciTech.

[5] Shi Q., Li F., Yeh S., Wang Y., Xin J. (2020). Physical stability of amorphous pharmaceutical solids: Nucleation, crystal growth, phase separation and effects of the polymers. Int. J. Pharm..

[6] Baghel S., Cathcart H., O’Reilly N.J. (2016). Polymeric Amorphous Solid Dispersions: A Review of Amorphization, Crystallization, Stabilization, Solid-State Characterization, and Aqueous Solubilization of Biopharmaceutical Classification System Class II Drugs. J. Pharm. Sci..

[7] Kalepu S., Nekkanti V. (2015). Insoluble drug delivery strategies: Review of recent advances and business prospects. Acta Pharm. Sin. B.

[8] Bhujbal S.V., Mitra B., Jain U., Gong Y., Agrawal A., Karki S., Taylor L.S., Kumar S., Zhou Q. (2021). Pharmaceutical amorphous solid dispersion: A review of manufacturing strategies. Acta Pharm. Sin. B.

[9] Zhang J., Liu Z., Wu H., Cai T. (2021). Effect of polymeric excipients on nucleation and crystal growth kinetics of amorphous fluconazole. Biomater. Sci..

[10] Lin X., Hu Y., Liu L., Su L., Li N., Yu J., Tang B., Yang Z. (2018). Physical Stability of Amorphous Solid Dispersions: A Physicochemical Perspective with Thermodynamic, Kinetic and Environmental Aspects. Pharm. Res..

[11] Lehmkemper K., Kyeremateng S.O., Bartels M., Degenhardt M., Sadowski G. (2018). Physical stability of API/polymer-blend amorphous solid dispersions. Eur. J. Pharm. Biopharm..

[12] Sahoo A., Kumar N.K., Suryanarayanan R. (2019). Crosslinking: An avenue to develop stable amorphous solid dispersion with high drug loading and tailored physical stability. J. Control. Release.

[13] Yao X., Neusaenger A.L., Yu L. (2021). Amorphous Drug-Polymer Salts. Pharmaceutics.

[14] Huang C., Powell C.T., Sun Y., Cai T., Yu L. (2017). Effect of Low-Concentration Polymers on Crystal Growth in Molecular Glasses: A Controlling Role for Polymer Segmental Mobility Relative to Host Dynamics. J. Phys. Chem. B.

[15] Sun Y., Zhu L., Wu T., Cai T., Gunn E.M., Yu L. (2012). Stability of Amorphous Pharmaceutical Solids: Crystal Growth Mechanisms and Effect of Polymer Additives. AAPS J..

[16] Powell C.T., Cai T., Hasebe M., Gunn E.M., Gao P., Zhang G., Gong Y., Yu L. (2013). Low-Concentration Polymers Inhibit and Accelerate Crystal Growth in Organic Glasses in Correlation with Segmental Mobility. J. Phys. Chem. B.

[17] Trasi N.S., Taylor L.S. (2012). Effect of Additives on Crystal Growth and Nucleation of Amorphous Flutamide. Cryst. Growth Des..

[18] Dahan A., Beig A., Lindley D., Miller J.M. (2016). The solubility–permeability interplay and oral drug formulation design: Two heads are better than one. Adv. Drug Deliv. Rev..

[19] Andronis V., Zografi G. (2000). Crystal nucleation and growth of indomethacin polymorphs from the amorphous state. J. Non-Cryst. Solids.

[20] Anwar J., Khan S., Lindfors L. (2015). Secondary Crystal Nucleation: Nuclei Breeding Factory Uncovered. Angew. Chem. Int. Ed..

[21] Xu S., Hou Z., Chuai X., Wang Y. (2020). Overview of Secondary Nucleation: From Fundamentals to Application. Ind. Eng. Chem. Res..

[22] Yao X., Liu Q., Wang B., Yu J., Aristov M.M., Shi C., Zhang G.G.Z., Yu L. (2022). Anisotropic Molecular Organization at a Liquid/Vapor Interface Promotes Crystal Nucleation with Polymorph Selection. J. Am. Chem. Soc..

[23] Vekilov P.G. (2004). Dense Liquid Precursor for the Nucleation of Ordered Solid Phases from Solution. Cryst. Growth Des..

[24] Alonzo D.E., Raina S., Zhou D., Gao Y., Zhang G.G.Z., Taylor L.S. (2012). Characterizing the Impact of Hydroxypropylmethyl Cellulose on the Growth and Nucleation Kinetics of Felodipine from Supersaturated Solutions. Cryst. Growth Des..

[25] Trasi N.S., Taylor L.S. (2012). Effect of polymers on nucleation and crystal growth of amorphous acetaminophen. CrystEngComm.

[26] Yao X., Huang C., Benson E.G., Shi C., Zhang G.G.Z., Yu L. (2019). Effect of Polymers on Crystallization in Glass-Forming Molecular Liquids: Equal Suppression of Nucleation and Growth and Master Curve for Prediction. Cryst. Growth Des..

[27] Miyazaki T., Aso Y., Yoshioka S., Kawanishi T. (2011). Differences in crystallization rate of nitrendipine enantiomers in amorphous solid dispersions with HPMC and HPMCP. Int. J. Pharm..

[28] Konno H., Taylor L.S. (2006). Influence of Different Polymers on the Crystallization Tendency of Molecularly Dispersed Amorphous Felodipine. J. Pharm. Sci..

[29] Wang Y., Wang Y., Cheng J., Chen H., Xu J., Liu Z., Shi Q., Zhang C. (2021). Recent Advances in the Application of Characterization Techniques for Studying Physical Stability of Amorphous Pharmaceutical Solids. Crystals.

[30] Huang C., Chen Z., Gui Y., Shi C., Zhang G.G.Z., Yu L. (2018). Crystal nucleation rates in glass-forming molecular liquids: D-sorbitol, D-arabitol, D-xylitol, and glycerol. J. Chem. Phys..

[31] Gui Y., Huang C., Shi C., Stelzer T., Zhang G.G.Z., Yu L. (2022). Polymorphic selectivity in crystal nucleation. J. Chem. Phys..

[32] Yao X., Benson E.G., Gui Y., Stelzer T., Zhang G.G.Z., Yu L. (2022). Surfactants Accelerate Crystallization of Amorphous Nifedipine by Similar Enhancement of Nucleation and Growth Independent of Hydrophilic–Lipophilic Balance. Mol. Pharm..

[33] Mapes M.K., Swallen S.F., Ediger M.D. (2006). Self-Diffusion of Supercooled *o*-Terphenyl near the Glass Transition Temperature. J. Phys. Chem. B.

[34] Swallen S.F., Ediger M.D. (2011). Self-diffusion of the amorphous pharmaceutical indomethacin near Tg. Soft Matter.

[35] Shi Q., Cai T. (2016). Fast Crystal Growth of Amorphous Griseofulvin: Relations between Bulk and Surface Growth Modes. Cryst. Growth Des..

[36] Shi Q., Wang Y., Xu J., Liu Z., Chin C.-Y. (2022). Fast crystal growth of amorphous nimesulide: Implication of surface effects. Acta Crystallogr. Sect. B Struct. Sci. Cryst. Eng. Mater..

[37] Yu L. (2016). Surface mobility of molecular glasses and its importance in physical stability. Adv. Drug Deliv. Rev..

[38] Sun Y., Zhu L., Kearns K.L., Ediger M.D., Yu L. (2011). Glasses crystallize rapidly at free surfaces by growing crystals upward. Proc. Natl. Acad. Sci. USA.

[39] Wang K., Sun C.C. (2019). Crystal Growth of Celecoxib from Amorphous State: Polymorphism, Growth Mechanism, and Kinetics. Cryst. Growth Des..

[40] Kestur U.S., Taylor L.S. (2010). Role of polymer chemistry in influencing crystal growth rates from amorphous felodipine. CrystEngComm.

[41] Taylor L.S., Zografi G. (1997). Spectroscopic Characterization of Interactions Between PVP and Indomethacin in Amorphous Molecular Dispersions. Pharm. Res..

[42] Kestur U.S., Lee H., Santiago D., Rinaldi C., Won Y.-Y., Taylor L.S. (2010). Effects of the Molecular Weight and Concentration of Polymer Additives, and Temperature on the Melt Crystallization Kinetics of a Small Drug Molecule. Cryst. Growth Des..

[43] Sato T., Taylor L.S. (2017). Acceleration of the crystal growth rate of low molecular weight organic compounds in supercooled liquids in the presence of polyhydroxybutyrate. CrystEngComm.

[44] Kestur U.S., Taylor L.S. (2013). Evaluation of the Crystal Growth Rate of Felodipine Polymorphs in the Presence and Absence of Additives As a Function of Temperature. Cryst. Growth Des..

[45] Zhang S., Britten J.F., Chow A.H.L., Lee T.W.Y. (2017). Impact of Crystal Structure and Polymer Excipients on the Melt Crystallization Kinetics of Itraconazole Polymorphs. Cryst. Growth Des..

[46] Shi Q., Zhang J., Zhang C., Jiang J., Tao J., Zhou D., Cai T. (2017). Selective Acceleration of Crystal Growth of Indomethacin Polymorphs by Low-Concentration Poly(ethylene oxide). Mol. Pharm..

[47] Tian B., Gao W., Tao X., Tang X., Taylor L.S. (2017). Impact of Polymers on the Melt Crystal Growth Rate of Indomethacin Polymorphs. Cryst. Growth Des..

[48] Madejczyk O., Kaminska E., Tarnacka M., Dulski M., Jurkiewicz K., Kaminski K., Paluch M. (2017). Studying the Crystallization of Various Polymorphic Forms of Nifedipine from Binary Mixtures with the Use of Different Experimental Techniques. Mol. Pharm..

[49] Kothari K., Ragoonanan V., Suryanarayanan R. (2014). The Role of Drug–Polymer Hydrogen Bonding Interactions on the Molecular Mobility and Physical Stability of Nifedipine Solid Dispersions. Mol. Pharm..

[50] Kothari K., Ragoonanan V., Suryanarayanan R. (2015). The Role of Polymer Concentration on the Molecular Mobility and Physical Stability of Nifedipine Solid Dispersions. Mol. Pharm..

[51] Mistry P., Suryanarayanan R. (2016). Strength of Drug–Polymer Interactions: Implications for Crystallization in Dispersions. Cryst. Growth Des..

[52] Mohapatra S., Samanta S., Kothari K., Mistry P., Suryanarayanan R. (2017). Effect of Polymer Molecular Weight on the Crystallization Behavior of Indomethacin Amorphous Solid Dispersions. Cryst. Growth Des..

[53] Mistry P., Mohapatra S., Gopinath T., Vogt F.G., Suryanarayanan R. (2015). Role of the Strength of Drug–Polymer Interactions on the Molecular Mobility and Crystallization Inhibition in Ketoconazole Solid Dispersions. Mol. Pharm..

[54] Shi Q., Zhang C., Su Y., Zhang J., Zhou D., Cai T. (2017). Acceleration of Crystal Growth of Amorphous Griseofulvin by Low-Concentration Poly(ethylene oxide): Aspects of Crystallization Kinetics and Molecular Mobility. Mol. Pharm..

[55] Zhang J., Shi Q., Tao J., Peng Y., Cai T. (2019). Impact of Polymer Enrichment at the Crystal–Liquid Interface on Crystallization Kinetics of Amorphous Solid Dispersions. Mol. Pharm..

[56] Zhang J., Shi Q., Guo M., Liu Z., Cai T. (2020). Melt Crystallization of Indomethacin Polymorphs in the Presence of Poly(ethylene oxide): Selective Enrichment of the Polymer at the Crystal–Liquid Interface. Mol. Pharm..

[57] Shi Q., Cheng J., Li F., Xu J., Zhang C. (2020). Molecular Mobility and Crystal Growth in Amorphous Binary Drug Delivery Systems: Effects of Low-Concentration Poly(Ethylene Oxide). AAPS PharmSciTech.

[58] Su Y., Yu L., Cai T. (2018). Enhanced Crystal Nucleation in Glass-Forming Liquids by Tensile Fracture in the Glassy State. Cryst. Growth Des..

[59] Powell C.T., Xi H., Sun Y., Gunn E., Chen Y., Ediger M.D., Yu L. (2015). Fast Crystal Growth in *o*-Terphenyl Glasses: A Possible Role for Fracture and Surface Mobility. J. Phys. Chem. B.

[60] Shi Q., Tao J., Zhang J., Su Y., Cai T. (2020). Crack- and Bubble-Induced Fast Crystal Growth of Amorphous Griseofulvin. Cryst. Growth Des..

[61] Powell C.T., Chen Y., Yu L. (2015). Fracture of molecular glasses under tension and increasing their fracture resistance with polymer additives. J. Non-Cryst. Solids.

[62] Thakore S.D., Akhtar J., Jain R., Paudel A., Bansal A.K. (2021). Analytical and Computational Methods for the Determination of Drug-Polymer Solubility and Miscibility. Mol. Pharm..

[63] Duan P., Lamm M.S., Yang F., Xu W., Skomski D., Su Y., Schmidt-Rohr K. (2020). Quantifying Molecular Mixing and Heterogeneity in Pharmaceutical Dispersions at Sub-100 nm Resolution by Spin Diffusion NMR. Mol. Pharm..

[64] Vasanthavada M., Tong W.-Q., Joshi Y., Kislalioglu M.S. (2005). Phase Behavior of Amorphous Molecular Dispersions II: Role of Hydrogen Bonding in Solid Solubility and Phase Separation Kinetics. Pharm. Res..

[65] Marsac P.J., Shamblin S.L., Taylor L.S. (2006). Theoretical and Practical Approaches for Prediction of Drug–Polymer Miscibility and Solubility. Pharm. Res..

[66] Marsac P.J., Li T., Taylor L.S. (2009). Estimation of Drug–Polymer Miscibility and Solubility in Amorphous Solid Dispersions Using Experimentally Determined Interaction Parameters. Pharm. Res..

[67] Tao J., Sun Y., Zhang G.G.Z., Yu L. (2009). Solubility of Small-Molecule Crystals in Polymers: D-Mannitol in PVP, Indomethacin in PVP/VA, and Nifedipine in PVP/VA. Pharm. Res..

[68] Sun Y., Tao J., Zhang G.G.Z., Yu L. (2010). Solubilities of Crystalline Drugs in Polymers: An Improved Analytical Method and Comparison of Solubilities of Indomethacin and Nifedipine in PVP, PVP/VA, and PVAc. J. Pharm. Sci..

[69] Tian Y., Booth J., Meehan E., Jones D.S., Li S., Andrews G.P. (2013). Construction of Drug–Polymer Thermodynamic Phase Diagrams Using Flory–Huggins Interaction Theory: Identifying the Relevance of Temperature and Drug Weight Fraction to Phase Separation within Solid Dispersions. Mol. Pharm..

[70] Pekamwar S., Kulkarni1 D., Gadade D. (2021). Accidental formation of eutectics during crystal engineering of lamotrigine with solubility advantage and drug release efficiency. Asian J. Pharm..

[71] Panzade P., Shendarkar G., Kulkarni D., Shelke S. (2021). Solid State Characterization and Dissolution Enhancement of Nevirapine Cocrystals. Adv. Pharm. Bull..

[72] Knopp M.M., Tajber L., Tian Y., Olesen N.E., Jones D.S., Kozyra A., Löbmann K., Paluch K.J., Brennan C.M., Holm R. (2015). Comparative Study of Different Methods for the Prediction of Drug–Polymer Solubility. Mol. Pharm..

[73] Potter C.B., Davis M.T., Albadarin A.B., Walker G.M. (2018). Investigation of the Dependence of the Flory–Huggins Interaction Parameter on Temperature and Composition in a Drug–Polymer System. Mol. Pharm..

[74] Tian Y., Jacobs E., Jones D.S., McCoy C.P., Wu H., Andrews G.P. (2020). The design and development of high drug loading amorphous solid dispersion for hot-melt extrusion platform. Int. J. Pharm..

[75] Panzade P.S., Shendarkar G.R., Kulkarni D.A. (2022). Hot Melt Extrusion: An Emerging Green Technique for the Synthesis of High-Quality Pharmaceutical Cocrystals. J. Pharm. Innov..

[76] Anderson B.D. (2018). Predicting Solubility/Miscibility in Amorphous Dispersions: It Is Time to Move Beyond Regular Solution Theories. J. Pharm. Sci..

[77] Xiang T.-X., Anderson B.D. (2013). Molecular Dynamics Simulation of Amorphous Indomethacin-Poly(Vinylpyrrolidone) Glasses: Solubility and Hydrogen Bonding Interactions. J. Pharm. Sci..

[78] Xiang T.-X., Anderson B.D. (2017). Molecular Dynamics Simulation of Amorphous Hydroxypropylmethylcellulose and Its Mixtures With Felodipine and Water. J. Pharm. Sci..

[79] Xiang T.-X., Anderson B.D. (2019). Effects of Molecular Interactions on Miscibility and Mobility of Ibuprofen in Amorphous Solid Dispersions With Various Polymers. J. Pharm. Sci..

[80] Gumaste S.G., Gupta S.S., Serajuddin A.T.M. (2016). Investigation of Polymer-Surfactant and Polymer-Drug-Surfactant Miscibility for Solid Dispersion. AAPS J..

[81] Tian B., Tang X., Taylor L.S. (2016). Investigating the Correlation between Miscibility and Physical Stability of Amorphous Solid Dispersions Using Fluorescence-Based Techniques. Mol. Pharm..

[82] Sharma J., Singh B., Agrawal A.K., Bansal A.K. (2021). Correlationship of drug-polymer miscibility, molecular relaxation and phase behavior of sipyridamole amorphous solid dispersions. J. Pharm. Sci..

[83] Medarevi’c D., Djuris J., Barmpalexis P., Kachrimanis K., Ibri´c S. (2019). Analytical and computational methods for the estimation of drug-polymer solubility and miscibility in solid dispersions development. Pharmaceutics.

[84] Iemtsev A., Hassouna F., Mathers A., Klajmon M., Dendisová M., Malinová L., Školáková T., Fulem M. (2020). Physical stability of hydroxypropyl methylcellulose-based amorphous solid dispersions: Experimental and computational study. Int. J. Pharm..

[85] Bochmann E.S., Neumann D., Gryczke A., Wagner K.G. (2019). Micro-scale solubility assessments and prediction models for active pharmaceutical ingredients in polymeric matrices. Eur. J. Pharm. Biopharm..

[86] Huang Y., Dai W.-G. (2014). Fundamental aspects of solid dispersion technology for poorly soluble drugs. Acta Pharm. Sin. B.

[87] Purohit H.S., Taylor L.S. (2015). Miscibility of Itraconazole–Hydroxypropyl Methylcellulose Blends: Insights with High Resolution Analytical Methodologies. Mol. Pharm..

[88] Luebbert C., Huxoll F., Sadowski G. (2017). Amorphous-Amorphous Phase Separation in API/Polymer Formulations. Molecules.

[89] Qian F., Huang J., Zhu Q., Haddadin R., Gawel J., Garmise R., Hussain M. (2010). Is a distinctive single Tg a reliable indicator for the homogeneity of amorphous solid dispersion?. Int. J. Pharm..

[90] Lodge T.P., Wood E.R., Haley J.C. (2006). Two calorimetric glass transitions do not necessarily indicate immiscibility: The case of PEO/PMMA. J. Polym. Sci. Part B Polym. Phys..

[91] Li N., Taylor L.S. (2016). Nanoscale infrared, thermal, and mechanical characterization of telaprevir-polymer miscibility in amorphous solid dispersions prepared by solvent evaporation. Mol. Pharm..

[92] Luebbert C., Klanke C., Sadowski G. (2018). Investigating phase separation in amorphous solid dispersions via Raman mapping. Int. J. Pharm..

[93] Yang R., Zhang G.G., Kjoller K., Dillon E., Purohit H.S., Taylor L.S. (2022). Phase separation in surfactant-containing amorphous solid dispersions: Orthogonal analytical methods to probe the effects of surfactants on morphology and phase composition. Int. J. Pharm..

[94] Konno H., Handa T., Alonzo D.E., Taylor L.S. (2008). Effect of polymer type on the dissolution profile of amorphous solid dispersions containing felodipine. Eur. J. Pharm. Biopharm..

[95] Ilevbare G.A., Liu H., Edgar K.J., Taylor L.S. (2013). Impact of Polymers on Crystal Growth Rate of Structurally Diverse Compounds from Aqueous Solution. Mol. Pharm..

[96] Ilevbare G.A., Liu H., Edgar K.J., Taylor L.S. (2012). Understanding Polymer Properties Important for Crystal Growth Inhibition—Impact of Chemically Diverse Polymers on Solution Crystal Growth of Ritonavir. Cryst. Growth Des..

[97] Alonzo D.E., Gao Y., Zhou D., Mo H., Zhang G.G., Taylor L.S. (2011). Dissolution and Precipitation Behavior of Amorphous Solid Dispersions. J. Pharm. Sci..

[98] Jackson M.J., Kestur U.S., Hussain M.A., Taylor L.S. (2016). Dissolution of Danazol Amorphous Solid Dispersions: Supersaturation and Phase Behavior as a Function of Drug Loading and Polymer Type. Mol. Pharm..

[99] Saboo S., Moseson D.E., Kestur U.S., Taylor L.S. (2020). Patterns of drug release as a function of drug loading from amorphous solid dispersions: A comparison of five different polymers. Eur. J. Pharm. Sci..

[100] Nunes P.D., Pinto J.F., Henriques J., Paiva A.M. (2022). Insights into the Release Mechanisms of ITZ:HPMCAS Amorphous Solid Dispersions: The Role of Drug-Rich Colloids. Mol. Pharm..

[101] Ting J., Tale S., Purchel A.A., Jones S.D., Widanapathirana L., Tolstyka Z.P., Guo L., Guillaudeu S., Bates F.S., Reineke T.M. (2016). High-Throughput Excipient Discovery Enables Oral Delivery of Poorly Soluble Pharmaceuticals. ACS Cent. Sci..

[102] Ting J.M., Porter III W.W., Mecca J.M., Bates F.S., Reineke T.M. (2018). Advances in polymer design for enhancing oral drug solubility and delivery. Bioconjugate Chem..

[103] Amponsah-Efah K.K., Mistry P., Eisenhart R., Suryanarayanan R. (2020). The Influence of the Strength of Drug–Polymer Interactions on the Dissolution of Amorphous Solid Dispersions. Mol. Pharm..

[104] Chen Y., Liu C., Chen Z., Su C., Hageman M., Hussain M., Haskell R., Stefanski K., Qian F. (2015). Drug–Polymer–Water Interaction and Its Implication for the Dissolution Performance of Amorphous Solid Dispersions. Mol. Pharm..

[105] Liu L., Chen L., Müllers W., Serno P., Qian F. (2022). Water-Resistant Drug–Polymer Interaction Contributes to the Formation of Nano-Species during the Dissolution of Felodipine Amorphous Solid Dispersions. Mol. Pharm..

[106] Ueda K., Hate S.S., Taylor L.S. (2020). Impact of Hypromellose Acetate Succinate Grade on Drug Amorphous Solubility and In Vitro Membrane Transport. J. Pharm. Sci..

[107] Sugihara H., Taylor L.S. (2018). Evaluation of pazopanib phase behavior following pH-induced supersaturation. Mol. Pharm..

[108] Schram C.J., Beaudoin S.P., Taylor L.S. (2015). Impact of Polymer Conformation on the Crystal Growth Inhibition of a Poorly Water-Soluble Drug in Aqueous Solution. Langmuir.

[109] Wang S., Liu C., Chen Y., Zhu A.D., Qian F. (2018). Aggregation of Hydroxypropyl Methylcellulose Acetate Succinate under Its Dissolving pH and the Impact on Drug Supersaturation. Mol. Pharm..

[110] Que C., Gao Y., Raina S.A., Zhang G.G.Z., Taylor L.S. (2018). Paclitaxel Crystal Seeds with Different Intrinsic Properties and Their Impact on Dissolution of Paclitaxel-HPMCAS Amorphous Solid Dispersions. Cryst. Growth Des..

[111] Qian K., Stella L., Jones D.S., Andrews G.P., Du H., Tian Y. (2021). Drug-rich phases induced by amorphous solid dispersion: Arbitrary or intentional goal in oral drug delivery?. Pharmaceutics.

[112] Saboo S., Mugheirbi N.A., Zemlyanov D.Y., Kestur U.S., Taylor L.S. (2019). Congruent release of drug and polymer: A “sweet spot” in the dissolution of amorphous solid dispersions. J. Control. Release.

[113] Chen Y., Wang S., Wang S., Liu C., Su C., Hageman M., Hussain M., Haskell R., Stefanski K., Qian F. (2016). Sodium Lauryl Sulfate Competitively Interacts with HPMC-AS and Consequently Reduces Oral Bioavailability of Posaconazole/HPMC-AS Amorphous Solid Dispersion. Mol. Pharm..

[114] Ueda K., Taylor L.S. (2021). Partitioning of surfactant into drug-rich nanodroplets and its impact on drug thermodynamic activity and droplet size. J. Control. Release.

[115] Raina S.A., Zhang G.G.Z., Alonzo D.E., Wu J., Zhu D., Catron N.D., Gao Y., Taylor L.S. (2015). Impact of Solubilizing Additives on Supersaturation and Membrane Transport of Drugs. Pharm. Res..

[116] Alhalaweh A., Bergstrom C.A.S., Taylor L.S. (2016). Compromised in vitro dissolution and membrane transport of multidrug amorphous formulations. J. Control. Release.

[117] Li N., Taylor L.S. (2018). Tailoring supersaturation from amorphous solid dispersions. J. Control. Release.

